# Evaluation of the clinical practice of aminoglycoside use in paediatric patients in Kenya: findings and implications for lower-middle income countries

**DOI:** 10.1093/jacamr/dlz087

**Published:** 2020-01-27

**Authors:** Elias Joseph Onyango, Faith Okalebo, Margaret Oluka, Rosaline Kinuthia, Loice Achieng, Brian Godman, Amanj Kurdi

**Affiliations:** 1 University of Nairobi, PO Box 19676 NAIROBI - 00202 KNH, Nairobi, Kenya; 2 Kenyatta National Hospital, PO Box 20723, Hospital Rd, Nairobi, Kenya; 3 Strathclyde Institute of Pharmacy and Biomedical Science, University of Strathclyde, 16 Richmond Street, Glasgow G1 1XQ, Scotland; 4 Division of Public Health Pharmacy and Management, School of Pharmacy, Sefako Makgatho Health Sciences University, Ga-Rankuwa, Pretoria, South Africa; 5 Division of Clinical Pharmacology, Karolinska Institute, Karolinska University Hospital Huddinge, Stockholm, Sweden; 6 Department of Pharmacology and Toxicology, College of Pharmacy, Hawler Medical University, Erbil, Iraq

## Abstract

**Objectives:**

To evaluate the practice of aminoglycoside use/monitoring in Kenya and explore healthcare worker (HCW) perceptions of aminoglycoside monitoring to identify gaps and opportunities for future improvements, given the low therapeutic index of aminoglycosides.

**Methods:**

This was a two-phase study whereby we reviewed patients’ medical records at Kenyatta National Hospital (October–December 2016) in Phase 1 and interviewed HCWs face to face in Phase 2. Outcome measures included describing and evaluating the practice of aminoglycoside use and monitoring and compliance to guidelines. Data were analysed using descriptive and inferential analysis.

**Results:**

Overall, out of the 2318 patients admitted, 192 patients (8.3%) were prescribed an aminoglycoside, of which 102 (53.1%) had aminoglycoside doses that did not conform to national guidelines. Aminoglycoside-related adverse effects were suspected in 65 (33.9%) patients. Monitoring of aminoglycoside therapy was performed in only 17 (8.9%) patients, with no therapeutic drug monitoring (TDM), attributed mainly to knowledge and skill gaps and lack of resources. Out of the 28 recruited HCWs, 18 (64.3%) needed training in how to perform and interpret TDM results.

**Conclusions:**

The practice of using and monitoring aminoglycosides was suboptimal, raising concerns around potential avoidable harm to patients. The identified gaps could form the basis for developing strategies to improve the future use of aminoglycosides, not only in Kenya but also in other countries with similar settings and resources.

## Introduction

Systemic aminoglycoside antibiotics (gentamicin, amikacin and tobramycin) are high-risk medicines.^1^ If not used and monitored appropriately, aminoglycosides may be associated with serious adverse effects including permanent hearing loss, vestibular toxicity and reversible nephrotoxicity.^2–4^ Children are more vulnerable to aminoglycoside toxicity due to reduced clearance.^5^ For aminoglycosides, serum creatinine concentrations are used to monitor possible nephrotoxicity and a baseline serum creatinine concentration should be obtained before initiating therapy. An increase in serum creatinine of greater than 25%–30% above the baseline, after ruling out other causes of nephrotoxicity, indicates aminoglycoside toxicity, requiring a switch to other treatment alternatives or intense monitoring.^3^

Despite the need for strategies to monitor its use, neither therapeutic drug monitoring (TDM) nor treatment protocols for aminoglycosides are currently available in Kenyatta National Hospital (KNH), the largest referral hospital in Kenya, apart from the national Basic Paediatric Protocol (BPP) for the management of critically ill paediatric patients.^6^ The pharmacy department in the hospital intends to introduce a TDM service. However, the opportunities and gaps for implementation of TDM of aminoglycosides at KNH are unknown. Consequently, the objectives of this study were to evaluate current prescribing and monitoring of aminoglycosides in the paediatric wards of KNH, including adherence to the national protocol, and secondly, to assess the willingness, understanding and knowledge of healthcare workers (HCWs) regarding TDM of aminoglycosides in KNH prior to introducing a monitoring service.

## Methods

The study consisted of two phases: the first phase was a medication-use review of aminoglycosides in paediatric patients over a 3 month period from October to December 2016 on the four general paediatric wards (GPWs) and the newborn unit (NBU) in KNH. This included evaluation of key processes in the medication-use cycle including prescribing, administration, monitoring, drug safety issues and patient outcomes. The second phase was structured interviews with HCWs.

### Study cohort and participants

The study cohort for Phase 1 was paediatric patients admitted to the GPWs/NBU of KNH during the study period who were on any aminoglycoside, with ages between 0 and 12 years. Patients were excluded if they were discharged before recruitment, not on any aminoglycosides during the period of the study, admitted to paediatric wards other than the GPWs or the NBU or were older than 12 years. Phase 2 included a structured interview of the HCWs in the GPWs and NBU.

Universal sampling was used. For Phase 1, all the files of patients meeting the inclusion/exclusion criteria were sampled and reviewed. The sampling was done within 48 h after the aminoglycoside was prescribed. Signed consent to collection of the data was given by the parents/guardians for children aged <7 years and both parental consent and child assent was obtained for children aged ≥7 years. For Phase 2, paediatrician specialists (*n *=* *40), senior house officers (*n *=* *15), pharmacists (*n *=* *4), nurses who were mainly responsible for the paediatric wards (*n *=* *6) and laboratory technicians (*n *=* *3) were contacted by phone as well as approached after completion of their ward rounds and asked to participate in the study.

### Sample size determination

In Phase 1, the sample size was calculated using the Cochran formula.^7^ Based on a previously reported aminoglycoside prevalence of 12.1%,^8^ a sample of 164 was estimated. To account for incomplete data, the calculated sample size was inflated by 10% to target a minimum of 181 patients.

### Data collection

A tool was designed to collect information on patient sociodemographic characteristics, medical history, medications prescribed, prescribing clinician and patient monitoring, developed by combining the WHO tool on investigating drug use,^9^ the NICE audit tool for antibiotics^10^ and a gentamicin-specific chart.^11^ Patient files were reviewed daily and data collection tools updated accordingly. As Phase 1 was an observation study, no blood samples were ordered/taken from the patients, rather we just recorded the serum creatinine from the patients’ files; creatinine level results dated within 48 h from the date of aminoglycoside prescribing were considered baseline creatinine levels and the others as follow-up creatinine levels.

In Phase 2, a questionnaire (available as Supplementary data at *JAC-AMR* Online) was developed by modifying a sample questionnaire for the Knowledge, Attitude and Practice (KAP) study on rational drug use by doctors.^12^ The data collection tools were piloted at one of the KNH paediatric wards by purposely sampling 10 patients on an aminoglycoside in Phase 1 and two HCWs in Phase 2.

### Study outcome measures

The study outcomes were describing and evaluating the practice of prescribing and monitoring aminoglycosides in paediatric wards, as well as determining the compliance to prescribing the correct aminoglycoside dosage based on the national BPP recommendations.^6^ Study covariates included patients’ ages, gender, weight and diagnosis, and the cadre of prescriber, antibiotic combinations, comedications, comorbidity and duration of aminoglycoside therapy. Monitoring of aminoglycoside therapy for adverse effects was assessed by monitoring serum creatinine levels, with nephrotoxicity defined as an increase of serum creatinine by >25% from the baseline creatinine level. Serum creatinine, rather than creatinine clearance, was used to assess nephrotoxicity due to two main reasons: first, it is the normal clinical practice to use serum creatinine to assess aminoglycoside-associated nephrotoxicity, not only in Kenya but also worldwide, including in the UK;^13^ secondly, serum creatinine is the only factor in the creatinine clearance formula that could change quickly, mainly in response to alteration in renal function during aminoglycoside therapy, compared with the other factors included in the creatinine clearance formula such as age and height of the child; therefore, any change in serum creatinine will be reflected directly in the creatinine clearance value. Hence serum creatinine provides similar information on patients’ renal function. Furthermore, in a setting with limited resources such as Kenya, it is more practical and feasible to use serum creatinine as it avoids the staff using a more complicated equation to calculate creatinine clearance, especially in view of the high workload pressure and limited number of staff in these resource-poor settings.

### Data analysis

Descriptive statistical analysis and inferential analysis were performed using the unpaired Student’s *t*-test for normally distributed continuous variables or the Mann–Whitney test for non-normally distributed continuous variables. The *χ*^2^ test was used for inferential data analysis for categorical variables. The sign rank test was used to determine whether the median doses complied with the recommendations in the guidelines. The level of significance was set at 0.05. The data were analysed using STATA version 13 software.

### Ethics

Study approval was obtained from the KNH/University of Nairobi Ethics Review Committee (approval number P563/07/2016).

## Results

### Phase 1

#### Baseline characteristics and prevalence of aminoglycoside use

A total of 2318 paediatric patients were admitted to the GPWs and NBU during the study period, of which 192 (8.3%) patients were prescribed aminoglycosides (Table 1). Out of the 192 eligible patients, 113 (58.9%) were male and 156 (81.2%) were aged ≤1 month (Table 1). The patient’s weight was documented in the majority of patients’ medical records (88%, *n *=* *169/192); however, the weight of a significant number of patients (52.3%, *n *=* *23/44) in the GPWs was not recorded.

**Table 1.** Baseline characteristics of the paediatric patients on aminoglycosides at the KNH

Characteristic	Ward, *n* (%)		
	GPW	NBU	total
Total admitted	1407 (60.7)	911 (39.3)	2318 (100)
Total enrolled	44 (3.1)	148 (16.2)	192 (8.3)
Sex			
male	22 (11.5)	91 (47.4)	113 (58.9)
female	22 (11.5)	57 (29.7)	79 (41.1)
Age category			
0–1 month	11 (5.7)	145 (75.5)	156 (81.2)
>1–24 months	19 (9.9)	3 (1.6)	22 (11.5)
>2–6 years	8 (4.2)	0 (0)	8 (4.2)
>6–12 years	6 (3.1)	0 (0)	6 (3.1)

Gentamicin was the most commonly used aminoglycoside (61.5%, *n *=* *118/192). The main indication for an aminoglycoside was neonatal sepsis (42.2%; *n *=* *81/192), followed by suspected infections with a presentation of respiratory distress (6.8%; *n *=* *13/192). A total of 39.6% (*n *=* *76/192) had two comorbidities and 33.3% (*n *=* *64/192) had one medical condition.

#### Number and types of medicines co-prescribed with aminoglycosides

The median total number of medicines per patient, including aminoglycosides, was 3 (range: 1–8), with 38% (*n *=* *73/192) on 2 medicines (Table 2). The median number of antibiotics per patient was 2 (range: 1–4) with the majority (84.9%; *n *=* *163/192) receiving 2 antibiotics at a time.

**Table 2.** Medications co-prescribed with aminoglycosides in paediatric wards of KNH

Ward, *n* (%)	Vitamins and supplements	Aminophylline	Gastrointestinal drugs	Analgesics	Anticonvulsants	Others^a^
GPW	13 (6.8)	1 (0.5)	5 (2.6)	9 (4.7)	4 (2.1)	22 (11.5)
NBU	47 (24.5)	22 (11.5)	11 (5.7)	4 (2.1)	9 (4.7)	23 (12.0)
Total	60 (31.3)	23 (12.0)	16 (8.3)	13 (6.8)	13 (6.8)	45 (23.5)

a The others were tetracycline eye ointment, saline nasal drops, anti-TB drugs, antiretrovirals, anti-opportunistic infection drugs and cardiovascular drugs.

#### Antibiotics co-administered with aminoglycoside

More than half of the patients (55.7%; *n *=* *107/192) were started on gentamicin/benzylpenicillin as the first-line antibiotic combination (Table 3). Amikacin/ceftazidime was the second most commonly prescribed (15.1%; *n *=* *29/192) combination. Only 48 (25%) patients were switched from one antibiotic combination to another. Out of these, 35.4% (*n *=* *17/48) were switched from gentamicin/benzylpenicillin to amikacin/ceftazidime, followed by a switch to oral antibiotics (16.7%; *n *=* *8/48). Fourteen patients (7.3%) had oral antibiotics administered with an aminoglycoside, with erythromycin being the most common (57.1%; *n *=* *8/14).

**Table 3.** Treatment of the most common infections in the paediatric wards of KNH

Initial antibiotic combination	Health problem, *n* (%)		
	severe pneumonia	suspected infections with presentation as respiratory distress	neonatal sepsis
Benzylpenicillin/gentamicin	5 (26.3)	22 (73.3)	41 (50.6)
Gentamicin only	4 (21)	0 (0)	2 (2.5)
Gentamicin/other antibiotics	0 (0)	0 (0)	1 (1.2)
Amikacin only	1 (5.3)	0 (0)	1 (1.2)
Amikacin/ceftazidime	1 (5.3)	6 (20)	12 (14.8)
Amikacin/meropenem	0 (0)	1 (3.3)	13 (16.0)
Amikacin/ceftriaxone	8 (42.1)	0 (0)	6 (7.4)
Amikacin/vancomycin	0 (0)	0 (0)	1 (1.2)
Amikacin/vancomycin/meropenem	0 (0)	1 (3.3)	0 (0)
Amikacin/other antibiotics	0 (0)	0 (0)	4 (4.9)
Total	19 (100)	30 (100)	81 (100)

Nephrotoxic drugs were concurrently administered with an aminoglycoside to 14 patients (7.3%), which included vancomycin (35.7%; *n *=* *5/14), IV furosemide (28.5%; *n *=* *4/14), NSAIDs (21.4%; *n *=* *3/14) and aciclovir (14.3%; *n *=* *2/14).

#### Dose and duration of aminoglycoside therapy in comparison with the national BPP treatment protocol

Although the prescribed mean gentamicin dose for neonates aged <7 days and weighing ≥2 kg was not significantly different from the recommended dose in the BPP protocol (3.4 versus 5 mg/kg/day; *P *=* *0.197), only 40.7% (*n *=* *24/59) of patients were prescribed the recommended dose of gentamicin of 5 mg/kg/day, with 37.3% (*n *=* *22/59) and 22.0% (*n *=* *13/59) being underdosed and overdosed, respectively. For neonates weighting <2 kg, the mean dose was significantly higher than the recommended dose (5 versus 3 mg/kg/day; *P *=* *0.006), with only 13.6% (*n *=* *3/22) of neonates prescribed the exact recommended dose and the majority (63.6%; *n *=* *14/22) being overdosed. Among those patients who were not prescribed the recommended aminoglycoside dose (*n *=* *49), 19 (38.8%) had neonatal sepsis.

For those aged >7 days, the mean gentamicin dose was not significantly different from the recommended dose (5.2 versus 7.5 mg/kg/day; *P *=* *0.068); however, only 14.3% (*n *=* *2/14) were prescribed the exact recommended dose, with the majority (57.1%, *n *=* *8/14) being underdosed, of which 75% (*n *=* *6/8) had neonatal sepsis. Similarly, the prescribed mean amikacin dose was not significantly different from the recommended dose of 15 mg/kg/day (*P *=* *0.552) for all patients; however, 33.0% (*n *=* *36/109) were underdosed.

In terms of duration of therapy, most of the patients (72.4%, *n *=* *139/192) were on an aminoglycoside for at least 72 h, with 39.6% (*n *=* *76/192) treated for at least 7 days. Slightly less than half (46.9%, *n *=* *90/192) of the patients did not complete the prescribed duration of therapy, mainly due to discharge (32.2%, *n *=* *29/90), change to other antimicrobials (30%, *n *=* *27/90) or death (28.9%, *n *=* *26/90).

#### Administration of aminoglycosides

All the patients received an aminoglycoside as an IV injection once daily. In all cases, gentamicin and amikacin were administered as a bolus infusion over 30 s. Half of the patients (49.5%, *n *=* *95/192) received all their daily doses of aminoglycoside within 1 h of the prescribed administration time; however, 16.7% (*n *=* *32/192) missed some aminoglycoside doses over the treatment course, of which the majority (59.4%, *n *=* *19/32) missed one dose and 28% (*n *=* *9/32) missed two doses. No reason was reported for the missed dose for the majority of cases (59.4%, *n *=* *19/32). Lack of an IV line was the reported reason in 10 cases (31.3%).

#### Monitoring of aminoglycoside therapy for adverse effects

Baseline and follow-up creatinine levels stratified by age categories are presented in Table S1. Baseline creatinine levels were recorded for the majority of the cases (68.8%, *n *=* *132/192), mostly for those aged 2 to 6 years (100%, *n *=* *9/9), with the least among those aged ≤30 days (63.5%, *n *=* *99/156). Among those patients with baseline creatinine level, 8.3% (*n *=* *11/132) had a documented baseline creatinine level above 120 μmol/L. Out of these 11 patients, aminoglycoside therapy was discontinued after one dose in 1 patient and after two doses in 2 patients. It was not documented whether the discontinuation was informed by the elevated creatinine level or other patient factors.

At least one follow-up creatinine level was measured in only 8.9% (*n *=* *17/192) of the patients, mostly in those aged 30 days to 24 months (28.6%, *n *=* *6/21), with the least among those aged 2 to 6 years (16.7%, *n *=* *1/6) (Table S1). Out of these, 11.8% (*n *=* *2/17) had follow-up creatinine levels above 100 μmol/L, while 29.4% (*n *=* *5/17) had an 11.7%–405% increase in their follow-up creatinine level compared with baseline values. Among patients who were on aminoglycoside therapy for ≥7 days (*n *=* *76), nine patients (11.8%) had a follow-up creatinine level at least twice a week. The median baseline creatinine level was 56 μmol/L (range: 17–239.7 μmol/L). The median follow-up creatinine level was 62.2 μmol/L (range: 26–128.3 μmol/L). An increase in baseline creatinine level of >30% was observed in one patient only, who received gentamicin/benzylpenicillin for 2 days then amikacin/ceftazidime for 6 days before being changed to meropenem for another 7 days.

#### Treatment outcome of paediatric patients on aminoglycoside therapy

The majority of the patients (68.3%, *n *=* *131/192) were successfully managed with the first-line antibiotic combination and were discharged without switching to other antimicrobials. Adverse drug effects were suspected in 33.9% (*n *=* *65/192) of patients. Fever resolved in all 53 patients who were febrile. Some patients (13.5%, *n *=* *26/192) died during the course of treatment.

#### Aminoglycoside-related potential adverse drug effects

Some of the suspected aminoglycoside-related adverse drug effects were nausea and vomiting (12.3%, *n *=* *8/65) and electrolyte imbalance (7.7%, *n *=* *5/65), of which 60% (*n *=* *3/5) had hyperkalaemia, 20% (*n *=* *1/5) had hypokalaemia and 20% (*n *=* *1/5) had hypernatraemia; 80% of them (*n *=* *4/5) were on gentamicin/benzylpenicillin. There was no significant difference in the incidence of suspected adverse drug effects between males and females [60% (*n *=* *39/65) versus 40% (*n *=* *26/65), *P *=* *0.790); however, a higher incidence was observed in the NBU (75.4%, *n *=* *49/65).

#### Summary of medication errors and identified gaps in using aminoglycosides

The most common type of medication error was a monitoring error (Table 4). This was followed by wrong dose and wrong time, respectively.

**Table 4.** Summary of medication errors in paediatric patients treated with aminoglycosides at KNH

Error	Ward, *n* (%)		
	GPW (*n *=* *44)	NBU (*n *=* *148)	total (*n *=* *192)
Omitted doses	3 (6.8)	29 (19.6)	32 (16.7)
Extra dose	2 (4.6)	22 (14.9)	24 (12.5)
Wrong time	19 (43.2)	78 (57.2)	97 (50.5)
Suspected adverse drug effects	16 (36.4)	49 (33.1)	65 (33.9)
Wrong combinations	8 (18.2)	6 (4.1)	14 (7.3)
Wrong dose errors			
underdose	11 (25)	60 (40.5)	71 (37.0)
overdose	4 (9)	27 (18.2)	31 (16.1)
total	15 (34.1)	87 (58.8)	102 (53.1)
Monitoring errors			
no baseline creatinine levels	1 (0.5)	59 (30.7)	60 (31.2)
no follow-up creatinine levels	36 (18.7)	138 (71.9)	174 (90.6)

### Phase 2: HCWs’ willingness, understanding and knowledge of TDM of aminoglycosides in KNH

#### Participant recruitment and response rate

**Figure 1.**
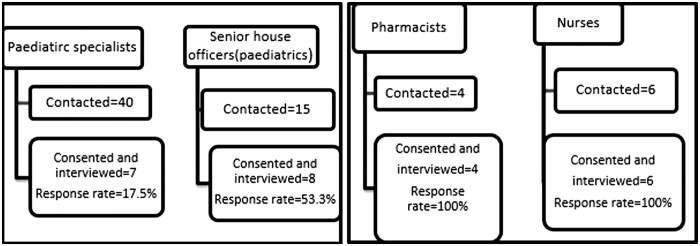
Flow chart of participant recruitment and response rate.

A total of 28 participants were recruited including 7 paediatrician specialists (response rate 17.5%; 7/40), 8 senior house officers (response rate 53.3%), 4 pharmacists (response rate 100%), 6 nurses (response rate = 100%) and 3 laboratory technologists (response rate = 100%) (Figure 1).

#### Baseline characteristics

The majority of the participants were female (64.3%, *n *=* *18/28), with a median age of 38.5 years (range: 26–74 years) and a median duration of practice of 12.5 years (range: 3–45 years).

#### Current practice for determining aminoglycoside doses

Only a minority of the study participants (17.9%, *n *=* *5/28) reported using existing paediatric protocols and guidelines in determining aminoglycoside doses, mostly pharmacists (50%, *n *=* *2/4) (Table 5). Almost half (46.4%, *n *=* *13/28) stated they determined the dose based on weight and age, mostly nurses (66.7%, *n *=* *4/6), followed by senior house officers (62.5%, *n *=* *5/8). Four respondents (14.3%) reported consideration of renal function tests when determining the dose, mostly pharmacists (50%, *n *=* *2/4). The majority (53.6%, *n *=* *15/28) were unsure about current monitoring practices, mainly laboratory technologists (66.7%, *n *=* *2/3); six (21.4%) stated it is not currently performed and seven (25%) reported using renal function tests as a method of monitoring aminoglycoside therapy, mainly senior house officers (50%, *n *=* *4/8) (Table 5).

**Table 5.** The current practice for aminoglycoside dosing and monitoring at KNH, staff involvement and need for TDM

	Paediatrician (*n *=* *7)	Senior house officer (*n *=* *8)	Pharmacist (*n *=* *4)	Nurse (*n *=* *6)	Laboratory technologist (*n *=* *3)	Total (*n *=* *28)
Aminoglycoside dosing based on existing guidelines? (Yes)	1 (14.3)	1 (12.5)	2 (50)	1 (16.7)	0 (0)	5 (17.9)
Aminoglycoside dosing based on weight and age? (Yes)	3 (42.9)	5 (62.5)	1 (25)	4 (66.7)	0 (0)	13 (46.4)
Aminoglycoside dosing based on renal and liver function? (Yes)	1 (14.3)	1 (12.5)	2 (50)	0 (0)	0 (0)	4 (14.3)
Is there a system for checking aminoglycoside dose? (Yes)	2 (28.6)	2 (25)	1 (25)	2 (33.3)	0 (0)	7 (25)
What is the current practice for monitoring aminoglycosides?						
not done	2 (28.6)	0 (0)	1 (25)	2 (33.3)	1 (33.3)	6 (21.4)
not sure	4 (57.1)	4 (50)	2 (50)	3 (50)	2 (66.7)	15 (53.6)
renal function test	1 (14.3)	4 (50)	1 (25)	1 (16.7)	0 (0)	7 (25)
Prior involvement in TDM? (Yes)	4 (57.1)	6 (75)	2 (50)	4 (66.7)	0 (0)	16 (57.1)
Have you ever requested for TDM? (Yes)	2 (28.6)	1 (12.5)	1 (25)	0 (0)	0 (0)	4 (14.3)
Do you need training on TDM? (Yes)	3 (42.9)	6 (75)	4 (100)	4 (66.7)	1 (33.3)	18 (64.3)

Values are presented as *n* (%).

The majority (53.6%, *n *=* *15/28) reported lack of a double-checking system before administration of an aminoglycoside, six (21.4%) were unsure and the remaining seven (25%) reported the existence of several such double-checking systems, mostly paediatricians (28.6%, *n *=* *2/7) (Table 5), of whom 57.1% (*n *=* *4/7) reported a review by the consultants during ward rounds, confirmation by nurses before administration (14.3%, *n *=* *1/28) and cross-checking by pharmacy staff before issuance of the drugs (28.6%, *n *=* *2/28).

#### Knowledge and experience of TDM for aminoglycosides

Sixteen respondents (57.1%) reported previous experience with ordering and performing TDM, mostly senior house officers (75%, *n *=* *6/8) (Table 5). Eighteen respondents (64.3%) reported their need for training on TDM, mostly pharmacists (100%, *n *=* *4/4), followed by nurses (66.7%, *n *=* *4/6) (Table 5). When the respondents were asked to list medicines they thought would need TDM, aminoglycoside was mentioned by half of them (*n *=* *14/28).

#### HCWs’ perception of barriers

**Figure 2.**
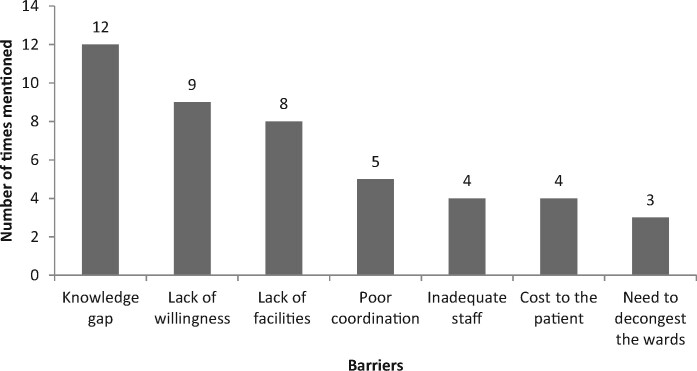
Opinion of the respondents on what can prevent effective implementation of a protocol on aminoglycoside dosing and monitoring in KNH.

When the respondents were asked about whether a treatment protocol on aminoglycoside dosing and monitoring is needed at KNH, all affirmed the need. When the study participants were asked about the content of such protocol, dose calculation and how to monitor patients were mentioned by 11 (39.3%), followed by information guidance around aminoglycoside-associated adverse drug effects (17.9%, *n *=* *5/28). In terms of the barriers, a knowledge gap was reported by 12 participants (42.9%) followed by a lack of willingness (32.9%, *n *=* *9/28) (Figure 2).

## Discussion

The study findings demonstrated several issues and concerns. Aminoglycoside doses for the majority of the patients (53.1%, *n *=* *102/192) did not conform to the recommendations in the national BPP. Furthermore, a reasonable number of patients (39.6%, *n *=* *76/192) received aminoglycosides for ≥7 days, which is more than the recommended duration in most current international and national guidelines.^14^ There were also major concerns regarding effective and safe monitoring of aminoglycosides, as indicated by the lack of baseline or follow-up serum creatinine. In addition, although TDM of aminoglycosides is not currently performed in KNH, the HCWs affirmed the need for TDM, but listed knowledge gaps, lack of willingness and poor coordination as potential barriers. There were also issues/limitations in the prescription and administration process of aminoglycosides.

The use of aminoglycosides in the paediatric wards was common, with an overall prevalence of 8.3%, lower though than the 12.1% reported by Erbay *et al.*^8^ and the 33.7% reported by Prajapati *et al*.^15^ This difference could be possibly attributed to concerns about aminoglycoside safety by paediatricians in KNH owing to the lack of TDM of aminoglycosides. However, differences in the study settings, and hence the treatment protocols, could also be a contributing factor.^8^^,^^15^

Of concern is that more than half (50.5%) of the patients did not receive their aminoglycoside doses within 1 h of the prescribed dose time, with some patients missing a dose for the entire day. This, combined with 30.3% of patients missing some aminoglycoside doses during the course of therapy, may contribute to decreased effectiveness and hence treatment failure, increasing mortality and resistance.

### Non-adherence to the Kenyan national protocol and implications

Incorrect dosing of aminoglycosides (either underdosing or overdosing) was one of the major and serious issues identified. However, it was difficult to establish the direct impact of this on patients’ clinical outcomes since the majority of these patients were prescribed an aminoglycoside in combination with other antibiotics. Consequently, the effect of aminoglycoside underdosing may not be detected immediately if the causative organism is responsive to the second antibiotic used in the correct dose; however, it can be a cause for concern if the organism is not responsive to the second antibiotic. Furthermore, among aminoglycoside-underdosed patients, the switching rate to another antibiotic was higher in those patients who received an aminoglycoside as monotherapy compared with an aminoglycoside as combination therapy. Switching of patients to other antibiotics could be an indicator of non-response due to underdosing. It was not clear though whether switching to other antibiotics was necessitated by non-response or by a change to more appropriate antibiotics.

Incorrect dosing could have been due to the failure to record the body weight for the majority of patients in the GPWs (52.3%, *n *=* *23/44). Although only 46.9% of patients received doses that conformed to the national paediatric protocol, this is higher than the 23% reported by Murgitroyd *et al.*^16^ and the 25% by Namazi *et al.*,^17^ indicating that the situation at KNH, although suboptimal, is better than at some other hospitals. However, some of the reasons listed for non-compliance by Murgitroyd *et al.*^16^ included knowledge gaps and time constraints, which were also highlighted in our study.

Fourteen (7.3%) patients were on other nephrotoxic drugs, lower than for Oliveira *et al.*^18^ and Gerlach *et al.*,^19^ who both reported 51%. This difference could be attributed to differences in the study populations since the latter two studies were in adults who, compared with paediatric patients, have higher chances of comorbidities and hence a higher likelihood of receiving nephrotoxic drug combinations. Only 1 patient out of 17 (5.9%) whose creatinine levels were followed up had nephrotoxicity; on the other hand, none of the patients whose creatinine levels were followed up had nephrotoxicity in the study by Rybak *et al.*,^20^ as compared with 1.6% in the study by Contopoulos-Ioannidis *et al*.^21^ In the current study, all patients were on a once-daily aminoglycoside dosing schedule, which may need reviewing, according to Rybak *et al.*,^20^ alongside the concurrent use of aminoglycosides with other nephrotoxins.^19^ Consequently, there is a need to minimize concurrent use of such drugs, if appropriate, or promote closer monitoring of patients. Electrolyte imbalance occurred in five (2.6%) patients. Hyponatraemia and derangement of potassium levels could be an indicator of nephrotoxicity. Hypernatraemia could have been a result of diarrhoea or vomiting.^22^

Currently, TDM at KNH is only undertaken for tacrolimus and cyclosporine but not for aminoglycosides. Consequently, aminoglycoside-related toxicity may not be detected early enough for swift intervention. The current limiting factors for performing TDM include a lack of aminoglycoside test kits and the contract agreement that dictates which medicines can be analysed, which does not include aminoglycosides. However, it is essential to address and overcome the barriers identified in this study before the introduction of such a TDM service to ensure effective and efficient uptake and implementation.^23–25^

### Identified gaps and potential future strategies and implications

The current study findings clearly indicate significant gaps in the practice of aminoglycoside use. These findings could form the basis for suggested strategies to improve the future use of aminoglycosides to optimize patients’ safety and quality of care. This could involve including aminoglycosides in future antimicrobial stewardship programmes (ASPs), which might include clear local protocols for prescribing, administrating and monitoring aminoglycosides, including signs of toxicity. These could incorporate clear instructions on how to calculate the correct aminoglycoside dose, initially and subsequently, based on patients’ age and weight.^26^ An online calculator or a mobile app could also be used to help calculate the correct initial aminoglycoside dose.^27^ One of the potential ASPs could be a standard patient chart for prescribing, administrating and monitoring of aminoglycosides with brief information including the maximum duration of 3–4 days.^13^ In the case of a longer treatment duration, advice should be sought from microbiologists, infectious disease physicians and antimicrobial pharmacists, including evidence-based guidelines on switching IV aminoglycosides to oral antibiotics when longer treatment is needed as longer treatment is associated with high toxicity.^28^^,^^29^ This is particularly important in KNH given that a longer aminoglycoside duration of ≥7 days was observed in nearly half of the patients (39.6%, *n *=* *76/192). It is also critical to have effective implementation strategies to ensure optimal uptake by clinicians, such as academic detailing, educational sessions and clinicians’ active engagement in the protocol design, development and implementation.^30–32^ These suggestions would improve the quality and safety of aminoglycoside use not only in Kenya but also in other African countries with similar settings.

### Strengths and limitations of the study

We are aware of several limitations. One of the challenges was incomplete medical records. Unavailability of TDM and minimal follow-up of creatinine levels was a limitation to monitoring patients for aminoglycoside toxicity. There could also be misclassification of patients for nephrotoxicity.

Whilst the questionnaire was not validated in this study, it has been designed and validated in other African countries with similar settings to Kenya.^12^ There was a low response rate by the paediatrician specialists, although it is unlikely that there are major differences in the perceptions of responders and non-responders since the main reason given for non-participation was time constraints. Overall, interviewing 28 participants appears to be an acceptable number in qualitative studies, with evidence indicating that level of thematic saturation is achieved in most cases after interviewing ≤16 participants.^33^

One strength was the use of both quantitative and qualitative approaches. The qualitative findings complemented/integrated the quantitative findings, the latter highlighting suboptimal practice in aminoglycoside dosing with reported potential aminoglycoside toxicity and adverse effects that indicate the need for and importance of having robust monitoring of aminoglycoside use, ideally through TDM. However, to perform TDM, it is crucial to understand the current situation at KNH in terms of facilitators and barriers for introducing TDM, most of which are provided by the qualitative findings.

### Conclusions

Aminoglycoside use appeared to be common among paediatric patients admitted to KNH. Adherence to the national treatment guideline was suboptimal and raises concerns around potential, avoidable harm to patients. Inadequate monitoring of paediatric patients on aminoglycosides is another matter of concern that requires urgent attention. The identified gaps highlight the need for potential strategies to improve the practice of aminoglycoside use including the development of ASPs. This situation analysis study has identified gaps and areas for quality improvement interventions, with the study findings serving as a baseline for any future interventions.

## Supplementary Material

dlz087_Supplementary_Data
