# Supplementary material for: Evaluation of the clinical practice of aminoglycoside use in paediatric patients in Kenya: findings and implications for lower-middle income countries
Source: JAC Antimicrob Resist. 2020 Jan 27;2(1):dlz087. doi: 10.1093/jacamr/dlz087 (PMC8210310; doi:10.1093/jacamr/dlz087)
Supplement: dlz087_Supplementary_Data [file dlz087_supplementary_data.zip › Supplementary_Data_I.docx]

**Supplementary data**

**Table S1. Baseline and follow-up creatinine level sub-grouped by age categories**

| Age categories | | | | | |
| --- | --- | --- | --- | --- | --- |
|  | 0-30 days (n=156) | 1 months-<2 years (n=21) | 2 -<6 years (n=9) | 6-12 years (n=6) | Total (n=192) |
| Baseline creatinine level recorded? (Yes) N (%) | 99 (63.5%) | 19 (90.1%) | 9 (100%) | 5 (83.3%) | 132 (68.8%) |
| At least one follow-up creatinine level recorded? (Yes) N (%) | 9 (5.8%) | 6 (28.6%) | 1 (11.1%) | 1 (16.7%) | 17 (8.9%) |
