# Supplementary material for: Evaluation of the clinical practice of aminoglycoside use in paediatric patients in Kenya: findings and implications for lower-middle income countries
Source: JAC Antimicrob Resist. 2020 Jan 27;2(1):dlz087. doi: 10.1093/jacamr/dlz087 (PMC8210310; doi:10.1093/jacamr/dlz087)
Supplement: dlz087_Supplementary_Data [file dlz087_supplementary_data.zip › Supplementary_Data_II.pdf]

APPENDIX B: INTERVIEW GUIDE FOR HEALTHCARE WORKERS

Dear colleague,

Kindly spare a few minutes to truthfully complete this questionnaire on aminoglycosides use in the paediatric wards of Kenyatta National Hospital.

**PART 1: DEMOGRAPHIC DETAILS**		
Gender	Male ☐	Age
	Female ☐	
Cadre	Pediatrician ☐	
	Senior House Officer ☐	
	Medical officer☐	
	Pharmacist ☐	
	Registered clinical officer ☐	
	Nurse ☐	
	Laboratory technologist ☐	
Duration in the profession		

**PART 2 CURRENT KNOWLEDGE AND PRACTICE**		
	Yes	No
Have you ever been involved in therapeutic drug monitoring before?		
Have you ever requested for TDM?		
If yes, where was it done		
For which drugs do you think TDM is critical in KNH?<br>1.		
2.		
3.		
4.		
5.		

KENYATTA NATIONAL HOSPITAL
APPROVED
23 AUG 2016
KNH/UoN-ERC
P.O. Box 20723-00202 NB.

INTERVIEW GUIDE

	Yes	No	Not sure
Do you need training on therapeutic drug monitoring			

How do you determine dose of aminoglycosides in paediatric patients in KNH currently?
How is monitoring of patients on aminoglycosides currently done in KNH?

	Yes	No
Is there a system for double checking the prescribed dose and preparation of aminoglycosides before administration to paediatric patients in KNH currently?		

PART 3: ATTITUDE AND BARRIERS TO IMPLEMENTATION OF PROTOCOL FOR AMINOGLYCOSIDES USE

	Yes	No
Do you think there is need for a protocol for aminoglycosides use in KNH?		

What do you think should be included in the protocol?
1.<br>2.
3.
4.
5.
What are the barriers that can prevent implementation of such protocol in KNH?
1.
2.
3.
4.
5.
6.

KENYATTA NATIONAL HOSPITAL
APPROVED
23 AUG 2016
KNH/UoN-ERC
P.O.Box 20723-00202 NRB.
