# Supplementary material for: Evaluation of the clinical practice of aminoglycoside use in paediatric patients in Kenya: findings and implications for lower-middle income countries
Source: JAC Antimicrob Resist. 2020 Jan 27;2(1):dlz087. doi: 10.1093/jacamr/dlz087 (PMC8210310; doi:10.1093/jacamr/dlz087)
Supplement: dlz087_Supplementary_Data [file dlz087_supplementary_data.zip › Reviewer report 1.docx]

**Reviewer report 1 (R0 – review of original submission)**

**PLEASE NOTE: JAC-AMR aims to publish your anonymous peer review comments with the final article for transparency. Please indicate in the box below whether you give your consent for us to publish your peer review comments for this article.**

Yes

**Synopsis**

**1. If the article contains original data, does the synopsis adequately reflect the manuscript as a whole, with the most important aspects described (of the introduction, methods and key results, and a balanced conclusion)? If NO, what is required to improve the synopsis? Please use the box below.**

Yes

**Introduction**

**2. Does the introduction provide the necessary background information for readers to understand the article? (e.g. does it describe the problem, current state of knowledge, etc.) If NO, what is required to improve it? Please use the box below**

Yes

**3. Is the research hypothesis and/or aims/objectives of the study clearly stated in the introduction? If NO, what is required to improve it? Please use the box below**

Yes

**4. Is there superfluous/unnecessary text in the introduction? (Only text that is required for readers to understand why the study has been done should be included). If YES, what text is superfluous and should be removed? Please use the box below**

No

Considering the answers above and any other comments/advice you may have, please indicate what is required to improve the Introduction:

**Methods**

**5. Were the methods used suitably robust and appropriate for the research question(s) and/or the aims/objectives of the study? If NO, the article should be rejected, but please advise the authors accordingly in the free-text box below and complete the review in full**

Yes

**6. Are the methods described in enough detail so the reader can understand what was done and interpret the results and conclusions appropriately?**

Yes

**7. Are the methods described in language that a reader who does not specialize in the area of the study/methods used will understand? For example, are the statistical approaches used described in language that non-statistician healthcare professionals will understand? If NO, please indicate what is required to improve the language in the methods section in the box below**

Yes

Considering the above and any other comments/advice you may have, please indicate other aspects of the methods section that could be improved in the free-text box below)

**Results**

**8. Given the methods used, are results described accurately, in enough detail and in an appropriate format to adequately inform the discussion section and the authors/readers conclusions? If NO, please indicate what is required to improve the results section in the box below**

Yes

**9. Based on what is described in the methods section, are data/results (that are likely to be available to the authors) missing from the current draft that you think should be presented to improve the manuscript?**

No

**10. Are Tables/Figures clear, required and do they add/complement the results text (they should not simply repeat what is in the text or vice versa)? If NO, please indicate what is required to improve the Tables/Figures in the box below**

Yes

Considering the above and any other comments/advice you may have, please indicate what is required to improve the results section:

**Discussion**

**11. Are the discussion and any conclusion(s) reasonable given the stated hypothesis/aims/objectives and results? If NO, please indicate how to improve the discussion in the box below**

Yes

**12. Is there superfluous/unnecessary text in the discussion? (Only text that directly relates to the results and understanding thereof in the context of existing published literature and the limitations of the study should be included.) If YES, please indicate what should be removed to improve the discussion in the box below**

No

**13. Is the Limitations sub-section adequate and reflective of the actual limitations of the study? ? If NO, please indicate how to improve the Limitations section in the box below**

Yes

Considering the above and any other comments/advice you may have, please indicate how to improve the discussion section:

**General**

**14. Is referencing throughout the manuscript satisfactory? (for instance are there statements for which you believe a supporting reference should be cited?) If NO, please indicate how to improve referencing in the box below**

Yes

**15. Is the language used (i.e. spelling, grammar, etc.) of high enough quality to be published as is? If NO, please indicate how to improve the language within the article in the box below**

Yes

**16. Are ethics/other approvals appropriate/adequate and satisfactorily described? ? If NO, please indicate how to improve this in the box below**

Yes

Considering the above and any other comments/advice you may have, please advise the authors how to improve these aspects of the article.

**Opinion and review articles only**

**17. Please advise the authors in the box below on how to improve their opinion or review article:**

|  **Recommendation** | |
| --- | --- |
|  | Accept (as is with no revisions) |
|  | Minor revision (with anticipation of subsequent acceptance if revisions are adequate) |
|  | Major Revision |
|  | Reject |

| **Would you be willing to review a revision of this manuscript?** | |
| --- | --- |
|  | Yes |
|  | No |

**Comments**

 Comments to the Author

If you have any additional comments to the authors about any aspect of the manuscript not covered in the structured review above, please place these in the box below. If you have no additional comments, please say “No additional comments” in box.

GENERAL COMMENTS:
This manuscript addresses the use of the aminoglycoside antibiotic, gentamicin in paediatric practice in a tertiary care facility in Kenya. It is well-written with achievable objectives, clear and concise results and a good discussion segment. However, there are few typographical and syntax errors which need to be corrected.
Please find below the observations:
1. Page 8, Lines 156 and 158 – Range to be written as 1- 4
2. Page 10, Line 206 – Consider replacing “” missing intravenous line” with "lack of intravenous line"
3. Page 11, Line 220 and 221 – Replace “ranged” to Range
4. Page 11, Lines 249-250 - Kindly clarify what is meant by a response rate of 17.5%. Does it imply that only two paediatricians responded ?
5. Page 11, Line 260 - Grouping together all respondents may not give us a good understanding of the study outcomes as there are differences in knowledge and level of practice between doctors, nurses and laboratory scientists. Is there a way of getting out the responses of the cadres separately?
6. Page 12, Lines 276-277 - Same comments as above. Nurses may not need to know how to order for or perform TDM
7. Page 14, Line 316 – Please add “with other antibiotics” after “combination”
8. Page 14, Line 334 - The sentence is best commenced with words - fourteen (14)
9. Page 15, Line 339 - Using 0.0% is not appropriate. Why not re-phrase like this...- none of the patients whose creatinine levels were followed up had nephrotoxicity in a study by ...... as compared to 1.6% found in....
10. Page 16, Line 386 -387- This assertion may not be right; kindly delete.
11. Page 17, Line 393 - Kindly write in full
