# Supplementary material for: Evaluation of the clinical practice of aminoglycoside use in paediatric patients in Kenya: findings and implications for lower-middle income countries
Source: JAC Antimicrob Resist. 2020 Jan 27;2(1):dlz087. doi: 10.1093/jacamr/dlz087 (PMC8210310; doi:10.1093/jacamr/dlz087)
Supplement: dlz087_Supplementary_Data [file dlz087_supplementary_data.zip › Reviewer report 2.docx]

**Reviewer report 2 (R0 – review of original submission)**

**PLEASE NOTE: JAC-AMR aims to publish your anonymous peer review comments with the final article for transparency. Please indicate in the box below whether you give your consent for us to publish your peer review comments for this article.**

Yes

**Synopsis**

**1. If the article contains original data, does the synopsis adequately reflect the manuscript as a whole, with the most important aspects described (of the introduction, methods and key results, and a balanced conclusion)? If NO, what is required to improve the synopsis? Please use the box below.**

Yes

Use of mixed design in the abstract needs to be expounded

include the review period

Outcome measures not clearly stated

What does monitoring refer here (TDM or safety?)

Be interesting to give statistics on the prevalence of aminoglycopsides use

**Introduction**

**2. Does the introduction provide the necessary background information for readers to understand the article? (e.g. does it describe the problem, current state of knowledge, etc.) If NO, what is required to improve it? Please use the box below**

Yes

**3. Is the research hypothesis and/or aims/objectives of the study clearly stated in the introduction? If NO, what is required to improve it? Please use the box below**

Yes

**4. Is there superfluous/unnecessary text in the introduction? (Only text that is required for readers to understand why the study has been done should be included). If YES, what text is superfluous and should be removed? Please use the box below**

No

Considering the answers above and any other comments/advice you may have, please indicate what is required to improve the Introduction:

However the study should give some statistics on AG use and monitoring in literature. little is given about other studies

What do the guidelines recommend for Kenya?

What does high risk medicines mean?

The introduction does not specifically address the problem under study (AG monitoring and use) focuses more on the outcomes and implementation

**Methods**

**5. Were the methods used suitably robust and appropriate for the research question(s) and/or the aims/objectives of the study? If NO, the article should be rejected, but please advise the authors accordingly in the free-text box below and complete the review in full**

Yes

**6. Are the methods described in enough detail so the reader can understand what was done and interpret the results and conclusions appropriately?**

No

**7. Are the methods described in language that a reader who does not specialize in the area of the study/methods used will understand? For example, are the statistical approaches used described in language that non-statistician healthcare professionals will understand? If NO, please indicate what is required to improve the language in the methods section in the box below**

Yes

Considering the above and any other comments/advice you may have, please indicate other aspects of the methods section that could be improved in the free-text box below)

The methods should  clearly

Describe the inclusion/exclusion criteria
Criteria for creatinine clearance and how it was measured
criteria to determine overdose and under dose for AG

criteria for the study outcomes (e.g over dose vs under dose)

Why were comparative tests done? Chi square etc

its not clear how the MIXED design was applied (quantitative and qualitative methods)

Why a sample of only children 0-12 years? not 16 years

How was the association of the ADR with the AG arrived at and graded as suspected? Naranjo scale or assumptions? Need to use WHO toxicity guidance /evidence to arrive at such claims

**Results**

**8. Given the methods used, are results described accurately, in enough detail and in an appropriate format to adequately inform the discussion section and the authors/readers conclusions? If NO, please indicate what is required to improve the results section in the box below**

Yes

**9. Based on what is described in the methods section, are data/results (that are likely to be available to the authors) missing from the current draft that you think should be presented to improve the manuscript?**

Yes

**10. Are Tables/Figures clear, required and do they add/complement the results text (they should not simply repeat what is in the text or vice versa)? If NO, please indicate what is required to improve the Tables/Figures in the box below**

Yes

Considering the above and any other comments/advice you may have, please indicate what is required to improve the results section:

how were the quantitative and qualitative results integrated?

**Discussion**

**11. Are the discussion and any conclusion(s) reasonable given the stated hypothesis/aims/objectives and results? If NO, please indicate how to improve the discussion in the box below**

Yes

**12. Is there superfluous/unnecessary text in the discussion? (Only text that directly relates to the results and understanding thereof in the context of existing published literature and the limitations of the study should be included.) If YES, please indicate what should be removed to improve the discussion in the box below**

No

**13. Is the Limitations sub-section adequate and reflective of the actual limitations of the study? ? If NO, please indicate how to improve the Limitations section in the box below**

Yes

Considering the above and any other comments/advice you may have, please indicate how to improve the discussion section:

**General**

**14. Is referencing throughout the manuscript satisfactory? (for instance are there statements for which you believe a supporting reference should be cited?) If NO, please indicate how to improve referencing in the box below**

Yes

**15. Is the language used (i.e. spelling, grammar, etc.) of high enough quality to be published as is? If NO, please indicate how to improve the language within the article in the box below**

Yes

**16. Are ethics/other approvals appropriate/adequate and satisfactorily described? ? If NO, please indicate how to improve this in the box below**

Yes

Considering the above and any other comments/advice you may have, please advise the authors how to improve these aspects of the article.

**Opinion and review articles only**

**17. Please advise the authors in the box below on how to improve their opinion or review article:**

|  **Recommendation** | |
| --- | --- |
|  | Accept (as is with no revisions) |
|  | Minor revision (with anticipation of subsequent acceptance if revisions are adequate) |
|  | Major Revision |
|  | Reject |

| **Would you be willing to review a revision of this manuscript?** | |
| --- | --- |
|  | Yes |
|  | No |

**Comments**

 Comments to the Author

If you have any additional comments to the authors about any aspect of the manuscript not covered in the structured review above, please place these in the box below. If you have no additional comments, please say “No additional comments” in box.

This is a well written paper.

The methods require more details; for example the eligibility criteria as well as criteria for determining outcomes are not clearly specified in the methods. The use of mixed study is inappropriately used and this should be revised. It may be necessary to describe how the children were recruited and when the measurements such as CrCl were taken and this should be standardized in the analysis. These results would benefit from sub-analysis of the use/monitoring of AG use by age classification and severity of disease. Where were the TDM services accessed if there were none at the hospital? There are a few grammatical errors in the paper. line 235-236 seems to have conflicting information (hyperkalemia and hypokalemia)
