# Supplementary material for: Evaluation of the clinical practice of aminoglycoside use in paediatric patients in Kenya: findings and implications for lower-middle income countries
Source: JAC Antimicrob Resist. 2020 Jan 27;2(1):dlz087. doi: 10.1093/jacamr/dlz087 (PMC8210310; doi:10.1093/jacamr/dlz087)
Supplement: dlz087_Supplementary_Data [file dlz087_supplementary_data.zip › Reviewer report 3.docx]

**Reviewer report 3 (R1 – review of revised submission)**

|  **Recommendation** | |
| --- | --- |
|  | Accept (as is with no revisions) |
|  | Minor revision (with anticipation of subsequent acceptance if revisions are adequate) |
|  | Major Revision |
|  | Reject |

| **Would you be willing to review a revision of this manuscript?** | |
| --- | --- |
|  | Yes |
|  | No |

**Comments**

 Comments to the Author

Thank you for the revised paper. It reads better. At this stage its not clear why serum creatinine and not creatinine clearance was used to assess toxicity due to AGs. The use of CrCl is more accurate than Scr. Various methods are in place to translate the Scr into CrCl. The levels of SCr can be affected a number of factors and may be misleading. This should be updated accordingly.
